# A novel method for treatment of Class III malocclusion in growing patients

**DOI:** 10.1186/s40510-017-0192-y

**Published:** 2017-12-11

**Authors:** Saad A. Al-Mozany, Oyku Dalci, Mohammed Almuzian, Carmen Gonzalez, Nour E. Tarraf, M. Ali Darendeliler

**Affiliations:** 1 Discipline of Orthodontics, Faculty of Dentistry, University of Sydney, Sydney, Australia; 2 Oxford University Hospitals, NHS Foundation Trust, Oxford, 0X3 9DU UK; 3 Eastman Dental Hospital, UCLH NHS Foundation Trust, London, UK

## Abstract

**Background:**

Management of Class III malocclusion is one of the most challenging treatments in orthodontics, and several methods have been advocated for treatment of this condition. A new treatment protocol involves the use of an alternating rapid maxillary expansion and constriction (Alt-RAMEC) protocol, in conjunction with full-time Class III elastic wear and coupled with the use of temporary anchorage devices (TADs). The aim of this study was to evaluate the dento-skeletal and profile soft tissue effects of this novel protocol in growing participants with retrognathic maxilla.

**Methods:**

Fourteen growing participants (7 males and 7 females; 12.05 ± 1.09 years), who displayed Class III malocclusions with retrognathic maxilla, were recruited. Pre-treatment records were taken before commencing treatment (T1). All participants had a hybrid mini-implant-supported rapid maxillary expansion (MARME) appliance that was activated by the Alt-RAMEC protocol for 9 weeks. Full-time bone-anchored Class III elastics, delivering 400 g/side, were then used for maxillary protraction. When positive overjet was achieved, protraction was ceased and post-treatment records were taken (T2). Linear and angular cephalometric variables were blindly measured by one investigator and repeated after 1 month. An error measurement (Dahlberg’s formula) study was performed to evaluate the intra-examiner reliability. A paired-sample *t* test (*p* < 0.05) was used to compare each variable from T1 to T2.

**Results:**

Treatment objectives were achieved in all participants within 8.5 weeks of protraction. The maxilla significantly protracted (SNA 1.87°± 1.06°; Vert.T-A 3.29± 1.54 mm *p* < 0.001), while the mandibular base significantly redirected posteriorly (SNB −2.03° ± 0.85°, Vert.T-B − 3.43± 4.47 mm, *p* < 0.001 and *p* < 0.05 respectively), resulting in a significant improvement in the jaw relationship (ANB 3.95°± 0.57°, *p* < 0.001; Wits 5.15± 1.51 mm, *p* < 0.001). The Y-axis angle increased significantly (1.95° ± 1.11°, *p* < 0.001). The upper incisors were significantly proclined (+ 2.98°± 2.71°, *p* < 0.01), coupled with a significant retroclination of the lower incisors (− 3.2°± 3.4°, *p* < 0.05). The combined skeletal and dental effects significantly improved the overjet (5.62± 1.36 mm, *p* < 0.001) and the soft tissue Harmony angle (2.75° ± 1.8°, *p* < 0.001).

**Conclusions:**

Class III elastics, combined with the Alt-RAMEC activation protocol of the MARPE appliance, is an efficient treatment method for mild/moderate Class III malocclusions. The long-term stability of these changes needs further evaluation.

## Background

The incidence of Class III malocclusions ranges from 0.8–12% [1–3]. The etiology of Class III malocclusions can be categorized as either genetic or environmental in origin [3]. The craniofacial characteristics of the Class III malocclusion may be attributed to both a positional and a dimensional disharmony of numerous components of the craniofacial skeleton involving the cranial base, the maxilla, and/or the mandible [4–6]. Ellis and McNamara [7], in their cephalometric sample of 302 adult participants with Class III malocclusions, found that 45.5% of their sample had maxillary retrusion.

Treatment modalities range from dentofacial orthopedic treatments using protraction facemasks [8] and camouflage orthodontic treatments to a combined orthodontic jaw surgery. The extra-oral protraction face mask (PFM) is the most efficient appliance for short- to long-term use [9–11]. Rapid maxillary expansion (RME), in conjunction with PFM, has been claimed to disrupt circummaxillary sutures, which in turn might enhance the skeletal effects [12]. By contrast, some evidence has suggested that RME provides no benefit to the outcomes of PFM [13].

An elaboration of the RME protocol, in which the maxilla is alternately expanded and constricted (Alt-RAMEC) in a weekly cycle, has been demonstrated to produce a more pronounced “disarticulation” effect that allows for a significant amount of maxillary protraction in a considerably reduced amount of time [14]. A well-designed randomized clinical trial demonstrated that PFM combined with the Alt-RAMEC protocol resulted in significant maxillary protraction (0.93 mm, 95% CI, − 1.65, − 0.20; *p* = 0.013) with minimal unwanted clockwise rotation of the mandible (*p* < 0.05) when compared with patients who underwent treatment with conventional PFM and RME [15]. A case-controlled clinical trial showed no statistically significant differences in the cephalometric variables among participants who had their facemask protraction commenced during an Alt-RAMEC phase when compared with those whose maxillary protraction started at the end of the Alt-RAMEC cycle [16].

The modern incorporation of skeletal anchorage into the discipline of orthodontics has led to its utilization in the orthopedic treatment of Class III malocclusions. The use of surgical plates has eliminated the need for the cumbersome part-time extra-oral headgear appliance, and the protraction is maintained full-time. A recent systematic review suggested that maxillary protraction anchored with a bone-anchorage device induces more maxillary advancement with minimal dental side effects when compared with tooth-anchored appliances [17]. Although efficient protraction of the maxilla has been confirmed following the use of surgical plates coupled with intermaxillary Class III elastics, their insertion is undertaken under general anesthesia, unlike temporary anchorage devices (TADs), which are usually placed under local anesthesia [18].

No previous study has investigated the effectiveness of the use of the Alt-RAMEC protocol in conjunction with TAD-supported Class III elastic wear for protraction of the maxilla. The aim of this study was to test the null hypothesis that this new treatment protocol will provide no statistically significant dento-skeletal and profile soft tissue changes.

## Methods

### Participants

The study was registered with the Australia New Zealand (ANZ) Clinical Trial Registry (ACTRN:12610000220066, Ethical approval Number: X10-010). All participants from the treatment waiting list of the Orthodontic Department at Sydney Dental Hospital were screened. Initially, 42 growing participants were identified with Class III malocclusions. Of these 42 selected participants, 14 (7 males and 7 females; 12.05 ± 1.09 years) met the inclusion criteria. As the study is a case series analytical study, no sample size calculation was undertaken. The inclusion criteria were:

- Participants at Cervical Vertebral Maturational (CVM) Stage 2 or 3 and
- Participants with clinically diagnosed retrognathic or hypoplastic maxilla, anterior crossbite, and dental Class III molars and canines.

Participants with previous orthodontic/orthopedic treatment or congenital abnormalities were excluded. All records (T1) were taken in the centric relation (CR) before commencing the intervention. A senior clinician (OD) re-examined the participants to confirm the inclusion criteria. Written informed consent was obtained from the parents or guardians.

### Treatment protocol

#### Appliance setup phase

**Fig. 1:**
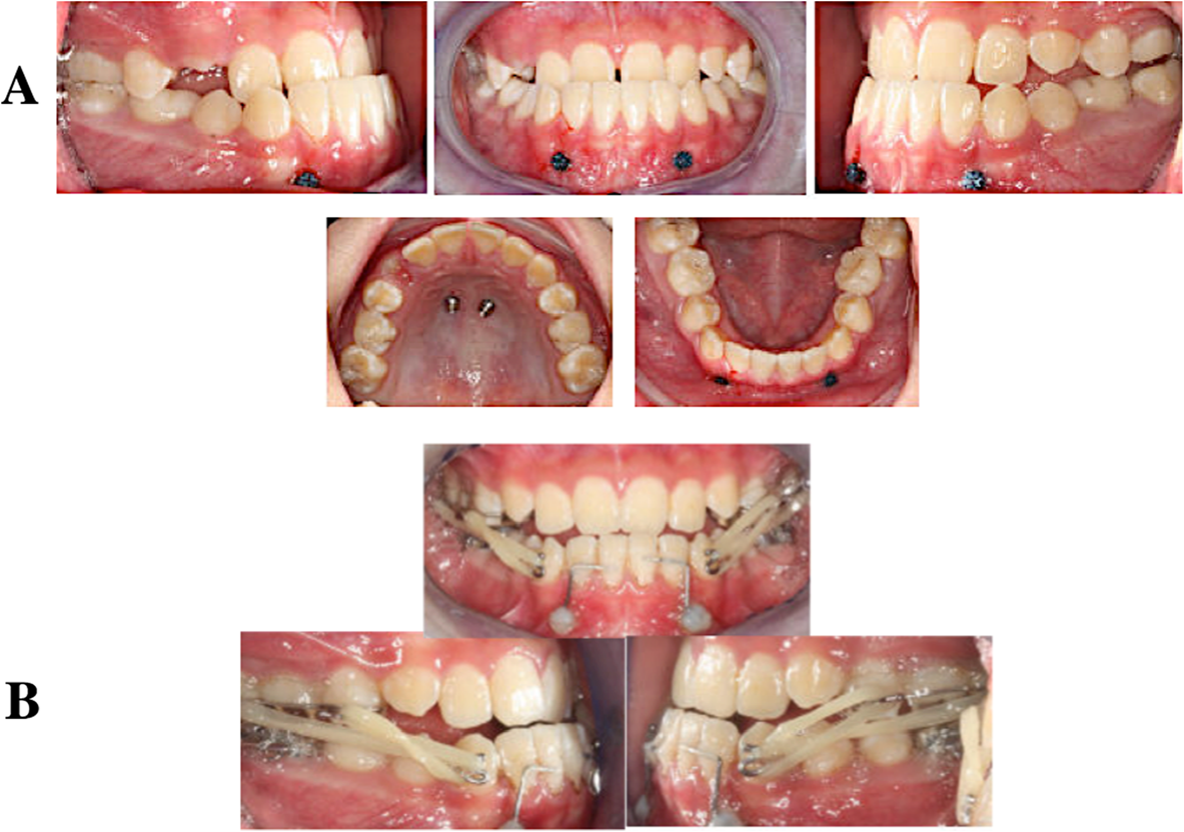
**a** Implant placement sites and **b** appliances loaded with two elastics per side

Each participant had four TADs inserted under local anesthesia (2% lignocaine with 1:80,000 adrenalin); two were para-medial palatal TADs and two were mandibular TADs that were inserted between the canine and the lateral incisor (Fig. 1a). Before placement of the TADs, the prospective implant site was swabbed with 0.12% chlorhexidine solution.

In the lower arch, self-drilling 1.6 × 6 mm Aarhus™ (MediconeG, American Orthodontics) TADs were placed at an approximately 30° apical angle. Insertion was complete when the head of the TAD was flush with the labial mucosa. The TADs chosen for the palatal placement were 2 × 9 mm Mondeal™ (GAC) TADs. The area of the palatal TAD placement was marked with a periodontal probe. Pilot 1.5-mm holes were then created using a surgical hand-piece (speed 800 rpm) under sodium chloride irrigation until engagement was achieved. The palatal TADs were then placed using a contra-angle handpiece (torque setting of 35 Ncm, speed 30 rpm). A minimum clearance of 5 mm between the two palatal TADs was chosen to enable the placement of the impression caps. Healing caps were then placed on the palatal TADs, and a 0.12% chlorhexidine mouth rinse was prescribed for daily use (Savacol, alcohol-free, Colgate).

One week later, molar bands were fitted around the lower first molars, and alginate impressions were then taken to construct a modified lingual arch (MLA). At the same visit, the palatal healing caps were removed and transfer impression copings were placed onto them for the subsequent transfer-coping polyvinylsiloxane (PVS) maxillary impressions. A medium-bodied PVS impression was injected around the transfer abutments, whereas the impression tray was filled with a heavy-bodied PVS. An impression of the maxillary arch was taken with the transfer abutments in place. After impression-taking, the laboratory mini-implant analogues were positioned on the impression transfer abutments. The three-dimensional relationships of the TADs in the oral cavity were thus duplicated on the plaster model.

#### Laboratory stage

**Fig. 2:**
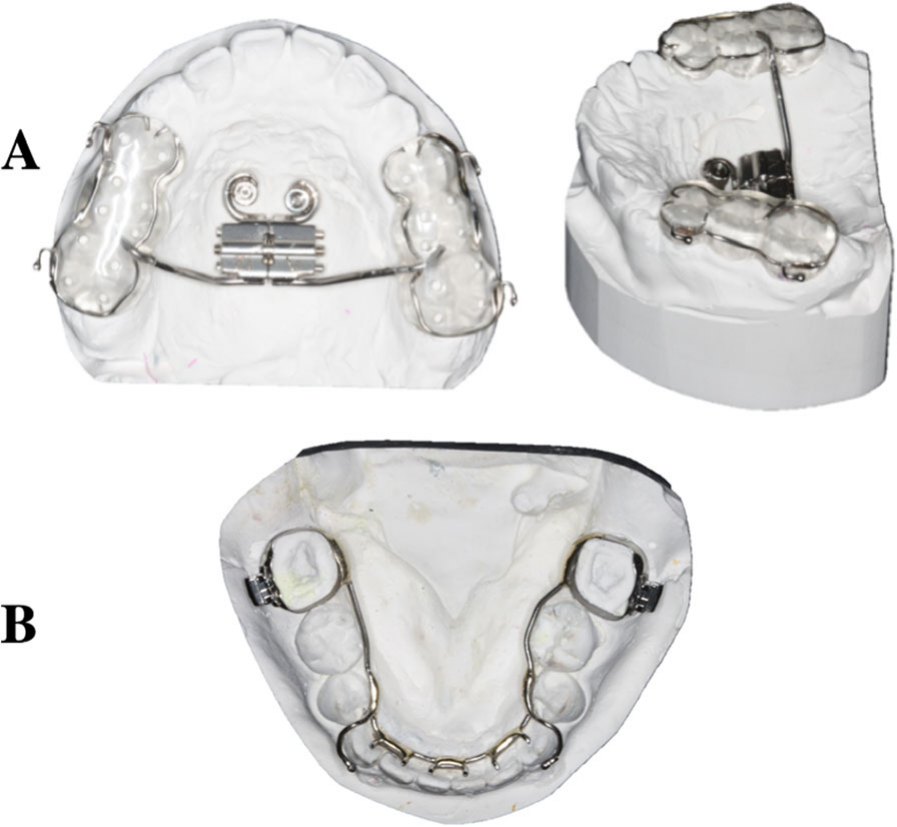
**a** Hybrid MARPE and **b** MLA appliance design

A hybrid mini-implant-assisted rapid maxillary expander (Hybrid MARPE), using a Hyrax-screw that produces 0.25 mm per quarter turn, was then constructed. Ball clasps (Romanium, Dentaurum) were embedded at the region of the first premolars and first molars (Fig. 2a). The Hybrid MARME was cemented with a glass ionomer cement (GIC) on day 28 of the TAD insertion.

The MLA was constructed from 1 mm romanium wire (Dentaurum, Australia) and cemented with GIC on day 28 after the TAD insertion. The lingual cleats that extended from the MLA were bonded onto the lingual surfaces of the anterior teeth with a composite resin to hold the lower arch as one unit (Fig. 2b).

#### Alt-RAMEC phase

The participant was instructed to expand the hybrid MARME by 1 mm/day for 7 days (2 turns in the morning and 2 turns in the evening). One week later, all participants presented for expansion assessment; if satisfactory, the participant was then instructed to constrict the maxilla by unwinding the hybrid MARME by 1 mm/day (2 turns in the morning and 2 turns in the evening) for 7 days. This cycle was repeated until week 9. After 9 weeks of alternating expansion and contraction, the mobility of the maxilla was subjectively and manually assessed. This was done by supporting the forehead and bridge of the participant’s nose with one hand and holding the maxillary incisors with the other. The maxilla was then moved in an anterior and posterior direction to detect the mobility of the maxilla. When sufficient mobility “disarticulation” was achieved, the second phase (the protraction phase) of treatment commenced.

#### Maxillary protraction phase

A 0.019″ × 0.025″ stainless steel (SS) wire was bent to fit passively into the crossheads of the lower TADs on both sides and was secured with a flowable composite to the labial surface of the lower incisors. Two full-time heavy intra-oral elastics per side, producing a total of 400 g/side, were prescribed. The participant was instructed to change the elastics once a day. One of these elastics ran in the long-closing Class III configuration, from the posterior ball clasps on the hybrid MARPE to the “S” hook. The other one ran in the short-closing Class III configuration, from the anterior hook on the hybrid MARPE to the MLA (Fig. 1b). This configuration was adopted to prevent counterclockwise rotation of the maxilla.

The participants were then assessed at 2-week intervals until a + 2-mm overjet was achieved. Once the overjet was corrected, the appliances were removed, and post-treatment records were then taken (T2).

### Cephalometric analysis

**Fig. 3:**
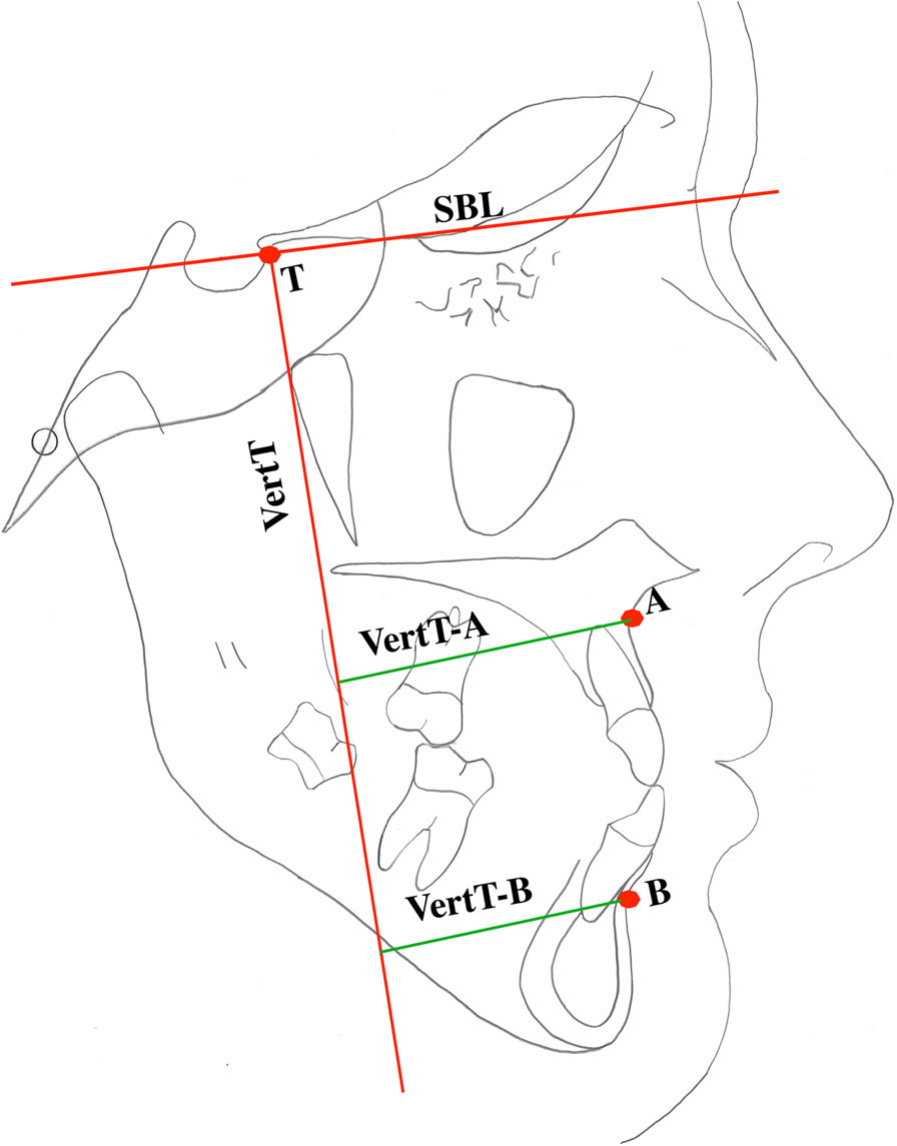
Horizontal skeletal measurements. Point T: The most superior point of the anterior wall of the sella turcica at the junction with tuberculum sellae, Vert T: Line perpendicular to SBL and passing through point T, Vert T-A: Horizontal distance traced from the perpendicular line from Vert T to point A, and Vert-B: Horizontal distance traced from the perpendicular line from Vert T to point B

One investigator blindly traced all the cephalograms using the Dolphin software. In addition to measuring the overjet changes as a primary outcome, the secondary outcomes included skeletal, dental, and soft tissue cephalometric measurements, as well as some of the recently described stable basicranial linear horizontal measurements [19] (Fig. 3 and table 1). The intra-examiner reliability was assessed by repeating all the cephalometric measurements after 1 month.

## Statistical analysis

The cephalometric data were analyzed statistically using the Statistical Package for Social Sciences (SPSS, ver. 17.0, SPSS Inc., Chicago, Illinois). The sample was normally distributed for most parameters, as determined using the Kolmogorov Smirnov test; hence, a paired-sample *t* test (*p* < 0.05) was used to compare each variable from T1 to T2. An error measurement (Dahlberg’s formula) study was performed to evaluate the intra-examiner reliability.

## Results

**Fig. 4:**
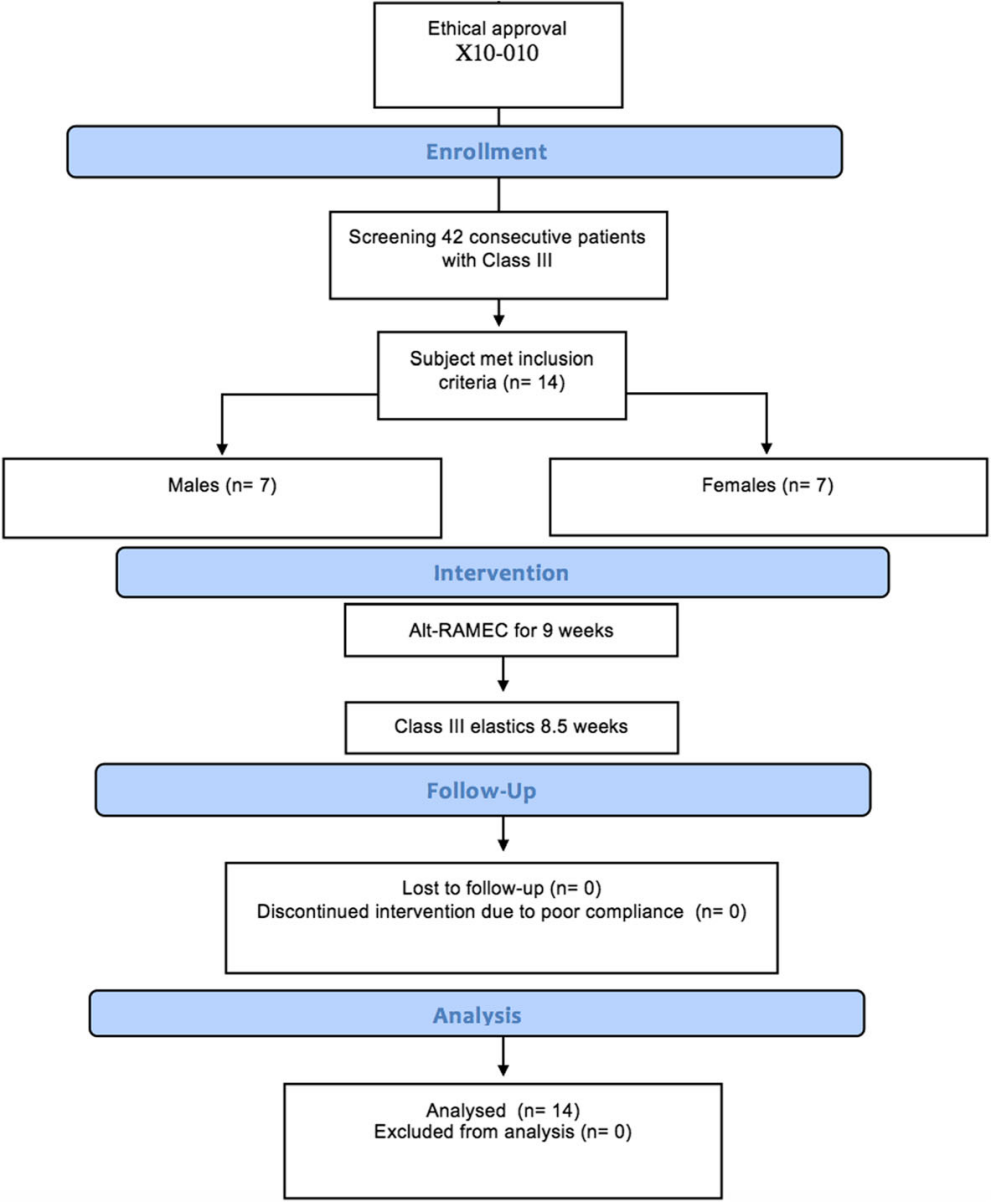
Trial flow chart

One mandibular TAD was lost but was replaced during the Alt-RAMEC phase. Another participant fractured the buccal attachment on the MLA, but this was repaired during elastic loading. Regardless, the aims of the treatment intervention were achieved in all participants over a period of 8.5 weeks of protraction (range 8–9 weeks) (Fig. 4).

Method errors were not statistically significant (*p* > 0.05), for both linear and angular measurements, at 0.98 mm and 0.87°, respectively. The pre-expansion (T1) and post-protraction (T2) cephalometric measurements are summarized in Table 1.

**Table 1:** Skeletal, dental, and soft tissue changes from T1 to T2

	T1	T2		T2-T1			
Variables	Mean	SD	Mean	SD	Mean	SD	*p* value
Anteroposterior changes							
SNA (°)	78.37	2.49	80.24	2.92	1.87	1.06	0.000
Vert. T-A (mm)	46.23	8.8	49.57	8.93	3.34	1.54	0.000
SNB (°)	82.11	3.19	80.09	3.53	− 2.02	0.85	0.000
Vert. T-B (mm)	39.57	14.69	36.14	12.95	− 3.43	4.47	0.013
ANB (°)	− 3.75	2.89	0.2	2.77	3.95	0.57	0.000
Wits appraisal (mm)	− 9.63	2.5	− 4.47	2.67	5.16	1.51	0.000
Vertical changes							
Mid-facial height (N-ANS) (mm)	52.27	2.99	54.95	2.35	2.68	1.53	0.447
Lower facial height (ANS-ME) (mm)	69.44	4.76	72.63	5.34	3.19	2.21	0.000
Upper facial height ratio (N-ANS/N-ME) (%)	44.3	1.88	43.13	1.91	− 1.17	1.21	0.003
Lower facial height ratio (N-ME/ANS-ME) (%)	55.67	1.99	56.87	1.91	1.2	1.24	0.003
Y-axis (°)	67.38	3.6	69.33	4.08	1.95	1.11	0.000
Dentoalveolar changes							
Upper incisors inclination (UI-SN)(°)	104.51	6.6	107.49	6.24	2.98	2.71	0.001
Lower incisors inclination (LI-MP)(°)	84.82	4.97	81.61	3.64	− 3.21	3.4	0.004
Inter-incisal angle (IIA) (°)	135.29	7.17	133.88	5.94	− 1.41	4.55	0.268
Overjet (OJ) (mm)	− 2.89	1.41	2.74	1.11	5.63	1.36	0.000
Overbite (OB) (mm)	1.57	1.92	0.36	1.46	− 1.21	1.89	0.033
Soft tissue profile changes							
Harmony (H) angle (n-me-ls)(°)	6.36	4.47	9.12	3.97	2.76	1.8	0.0001

At the skeletal level, both angular (Sella-Nasion to A (SNA) 1.87± 1.06 mm) and linear (Vert.T-A 3.34 ± 1.54 mm) measurements of the anteroposterior position of the maxilla showed a significant protraction (*p* < 0.001). Similarly, the mandible position was significantly improved (Vert.T-B − 3.43± 4.47 mm, *p* < 0.05; Sella-Nasion to B (SNB) − 2.02 ± 0.85, *p* < 0.001). A marked improvement was evident in the ANB angle (3.95° ± 0.57°, *p* < 0.001) and Wits measurement (5.16± 1.5 mm, *p* < 0.001). The significant increase in the Y-axis (1.95° ±1.22°, *p* < 0.001), coupled with a significant increase in the lower third (ANS-Me) of 3.19± 2.2 mm (*p* < 0.001), indicated a clockwise rotation of the mandible. However, no significant increase was noted in the middle facial height (N-ANS) (0.32± 1.53 mm, *p* = 0.45).

At the dental level, the upper incisors proclined significantly (UI-PP = 2.98° ± 2.71°, *p* < 0.005) coupled with a significant retroclination of the lower incisors (LI-MP = 3.2°± 3.4°, *p* < 0.05). The combined dental and skeletal changes led to a significant improvement in the overjet and overbite, at 5.62 ± 1.36 mm (*p* < 0.001) and − 1.21± 1.89 mm (*p* < 0.05), respectively.

Furthermore, cephalometric soft tissue profile analysis showed a significant increase in the H angle, at 2.76± 1.8 (*p* < 0.001).

## Discussion

The recommended starting age for maxillary protraction therapy for a good orthopedic effect is the prepubertal stage [20–23]. Nevertheless, the participants in this study (aged 12.05 ± 1.09 years) responded positively, with a mean treatment time of approximately 8.5 weeks.

The Alt-RAMEC protocol was utilized to produce a more pronounced disarticulation of the maxilla than can be obtained using conventional maxillary expansion [14]. The mean maxillary protraction was significantly greater than the outcomes reported in the previous literature [21, 24, 25]. This could be attributed to a combination of the disarticulation effect of the Alt-RAMEC protocol and/ or the full-time utilization of the heavy Class III elastics which were partially tooth-bone-anchored. Similarly, the anteroposterior mandibular position was significantly improved secondary to the intervention, again probably due to the full-time utilization of the Class III elastics. The argument might be made that the changes in the SNB and therefore ANB were surpassed as a result of the elimination of mandibular functional displacement secondary to the intervention however the main aim of our class III correction was to improve the maxillary position nevertheless taking records at the RCP could induce another inherent pseudo-increase in the facial height.

A posterior rotation of the mandible and an increase in the anterior facial height are common treatment biomechanical effects of the PFM treatment [21, 25–27]. Similar changes were observed in this study in the form of significant increases in the lower facial height and Y-axis.

The maxillary protraction protocol partially utilized the dentition for the transmission of the forces to the underlying skeletal structures, including the maxilla and the mandible. This led to the unwanted effects represented by proclination of the upper incisors and retroclination of the lower incisors, as reported in other studies [28, 29]. Therefore, correction of the malocclusion was due to the combination of skeletal and dentoalveolar effectst.

**Fig. 5:**
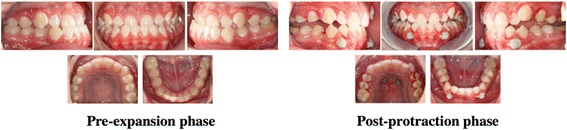
Records for patient 5

The skeletal and dentoalveolar changes observed in our study resulted in an overall normalization of the unesthetic facial concavity. This was seen as a significant reduction in the H angle of these participants. For a clinical demonstration, the treatment records are presented for one of the participants enrolled in this study (Fig. 5).

### Limitations of the study and future research

An argument can be made that the wide standard deviation of SNA angles could increase the level of uncertainty. This might be attributed to individual variations in response to the treatment and/or errors in tracing.

One of the aims of using TADs in our treatment protocol was to reduce the unwanted dentoalveolar side effects; however, proclination of the upper incisors and retroclination of the lower incisors were unavoidable. This could be a result of the inherent flexibility of the vertical arms that connect the lower TADs to the mandibular incisors, as this may have allowed wire flexion under the effect of the heavy inter-maxillary elastics, thereby allowing for retroclination of the lower incisors. Similarly, the arms that connect the palatal TADs to the acrylic pads of the hybrid MARPE may have flexed under the protractive effect of the Class III elastics, allowing for proclination of the maxillary incisors.

One of the difficulties in using this treatment protocol is the delicateness of implant appliance placement, as the slightest error in appliance impression/construction makes it difficult to issue the expander with palatal TADs. An alternative would be to design a new hybrid MARPE system that would permit the cementation of the expander with hooks for class III elastic placement first, followed by insertion of the TADs. Another drawback of this novel treatment approach is participant compliance with performing the expansion and constriction of the maxilla and the daily interchange of the elastics. Future developments may involve an expander that expands and contracts itself, as per a particular protocol, plus the development of intra-oral nickel titanium springs to minimize the participant’s compliance. Alternatively, magnets can be used to provide the protractive forces.

The authors acknowledge that the sample size of this study is too small to comment on the validity of the use of this novel approach in treating Class III malocclusion compared to other established methods. A future direction of the study would be to compare this treatment modality with other treatment approaches using a long-term randomized clinical trial.

## Conclusions

Bone-anchored Class III protraction using MARPE and miniscrew supported lower lingual arch and Alt-RAMEC protocol, is an efficient first phase treatment for Class III malocclusions. Correction was achieved through a combination of skeletal, and dentoalveolar effects. However, a long-term randomized clinical trial with a larger sample size is recommended for verification.
